# Study on the Influence of CaO on the Electrochemical Reduction of Fe_2_O_3_ in NaCl-CaCl_2_ Molten Salt

**DOI:** 10.3390/molecules28248103

**Published:** 2023-12-15

**Authors:** Hui Li, Lingyue Song, Jinglong Liang, Dongxing Huo, Weigang Cao, Chang Liu

**Affiliations:** 1 Key Laboratory of Modern Metallurgical Technology, Ministry of Education, College of Metallurgy and Energy, North China University of Science and Technology, Tangshan 063210, China; 2 College of Mechanical Engineering, North China University of Science and Technology, Tangshan 063210, China

**Keywords:** molten salt, electrochemical, Fe_2_O_3_-CaO

## Abstract

The presence of calcium-containing molten salts in the electrolysis of oxides for metal production can lead to the formation of CaO and, subsequently, the generation of intermediate products, affecting the reduction of metals. To investigate the impact of CaO on the reduction process, experiments were conducted using a Fe_2_O_3_-CaO cathode and a graphite anode in a NaCl-CaCl_2_ molten salt electrolyte at 800 °C. The electrochemical reduction kinetics of the intermediate product Ca_2_Fe_2_O_5_ were studied using cyclic voltammetry and *I*-*t* curve analysis. The phase composition and morphology of the electrolysis products were analyzed using XRD, SEM-EDS, and XPS. The experimental results demonstrate that upon addition of CaO to the Fe_2_O_3_ cathode, Ca_2_Fe_2_O_5_ is formed instantly in the molten salt upon the application of an electrical current. Research conducted at different voltages, combined with electrochemical analysis, indicates that the reduction steps of Ca_2_Fe_2_O_5_ in the NaCl-CaCl_2_ molten salt are as follows: Ca_2_Fe_2_O_5_ ⟶ Fe_3_O_4_ ⟶ FeO ⟶ Fe. The presence of CaO accelerates the electrochemical reduction rate, promoting the formation of Fe. At 0.6 V and after 600 min of electrolysis, all of the Ca_2_Fe_2_O_5_ is converted into Fe, coexisting with CaCO_3_. With an increase in the electrolysis voltage, the electrolysis product Fe particles visibly grow larger, exhibiting pronounced agglomeration effects. Under the conditions of a 1 V voltage, a study was conducted to investigate the influence of time on the reduction process of Ca_2_Fe_2_O_5_. Gradually, it resulted in the formation of CaFe_3_O_5_, CaFe_5_O_7_, FeO, and metallic Fe. With an increased driving force, one gram of Fe_2_O_3_-CaO mixed oxide can completely turn into metal Fe by electrolysis for 300 min.

## 1. Introduction

The traditional method of iron ore reduction and smelting relies on carbon thermal reduction, a process that emits a significant amount of greenhouse gases. This poses a substantial challenge to achieving carbon neutrality and peak carbon goals [1,2]. Consequently, there is widespread interest in seeking a more environmentally friendly and efficient method for iron production. Hydrogen, as the most promising clean energy source, has garnered attention as a reducing agent in metal smelting processes [3]. Its utilization in metal production is considered an effective pathway toward achieving green and sustainable development [4,5,6,7,8,9]. Furthermore, electrochemical technologies driven by low-carbon electrical energy, generated from renewable sources such as wind energy [10], nuclear energy [11], and solar energy [12], play a vital role in energy conversion. Therefore, electrochemical metallurgy, which relies on these technologies, has become a key element in the steel industry’s efforts to reduce carbon emissions [13,14,15].

The Molten Oxide Electrolysis (MOE) method involves the reduction of iron oxides to liquid metallic iron, along with the release of O_2_, in a molten oxide system at high temperatures, using an inert anode. However, due to the elevated process temperatures, some metals can be corroded during the anodic polarization process, making the selection of appropriate inert anodes a critical factor limiting their development [16,17]. The FFC Cambridge Process [18] proposes the use of solid TiO_2_ as the cathode in a CaCl_2_ molten salt system to electro-deoxidize and produce metallic titanium. In comparison to the MOE method, this process achieves the direct electrolytic production of metals and alloys at lower temperatures ranging from 800 °C to 850 °C [19,20]. The use of solid metal oxides as cathodes in chloride molten salts for a direct electrochemical reduction to produce elemental metals has been applied in the extraction of various metals, including Fe [21,22], Ti [23], Cr [24,25], Al [26], V [27,28], Se [29], titanium-based [30,31,32,33,34], aluminum-based alloys [35,36], high-entropy alloys [37], etc. Research has shown that the addition of a small amount of CaO to CaCl_2_ can significantly increase the rate of reduction and deoxidation [38,39,40]. Therefore, it is believed that the primary factor influencing the reduction process in CaCl_2_ molten salt is CaO. This is due to the fact that CaO dissolves in CaCl_2_ molten salt and enables O^2−^ ion transport under electrolytic conditions [41]. During electrolysis, Ca^2+^ ions can combine with nearby O^2−^ ions to form CaO, which can then react with metal oxides to produce intermediate phases that are subsequently reduced during the electrolysis process [42,43,44,45,46,47,48,49]. In the electrolytic production of metallic titanium in a CaCl_2_-NaCl mixture, CaO reacts with TiO_2_ to form an intermediate phase, CaTiO_3_. The use of sintered CaTiO_3_ as a precursor material has been found to reduce the electrolysis time [50,51]. However, in experiments involving the electrolytic synthesis of SiC in molten CaCl_2_, the interaction between SiO_2_ and CaO can lead to the formation of intermediate phases that are difficult to remove, thereby hindering the progress of the reaction [52]. Therefore, the influence of CaO on the formation of intermediate products and the reduction process is a topic that warrants further research.

To investigate the influence of intermediate phases generated during the electrolytic production of metals in CaCl_2_-containing molten salts, this study focuses on the reduction of metallic iron (Fe). Constant voltage electrolysis experiments were conducted using Fe_2_O_3_-CaO as the cathode in a NaCl-CaCl_2_ mixed molten salt system. The research examined the effects of electrolysis voltage and electrolysis time on the reduction behavior of Fe_2_O_3_-CaO. Through the study of the formation of the intermediate product Ca_2_Fe_2_O_5_ and its impact on the reduction process, in conjunction with electrochemical analysis methods, the electrode re-action mechanism was elucidated. This research aims to provide a theoretical basis for the preparation of other metals in calcium-containing molten salts.

## 2. Results and Discussion

### 2.1. Impact of Electrolysis Voltage

**Figure 1:**
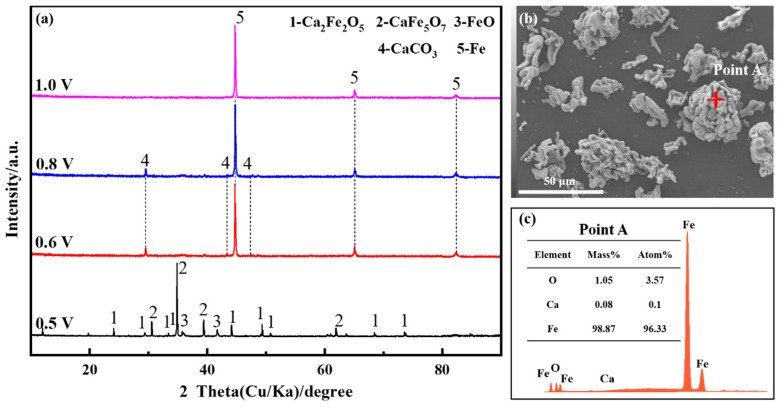
Fe_2_O_3_-CaO in NaCl-CaCl_2_ molten salt at different voltages for 600 min; (**a**) XRD patterns and (**b**,**c**) SEM and EDS image at 1.0 V for the constant cell voltage electrolysis.

Voltage, acting as the driving force in the electrolysis process, exerts control over the formation of the final products and determines the thermodynamic conditions. The constant cell voltage electrolysis experiment was carried out at 800 °C by applying different voltages (0.5 V, 0.6 V, 0.8 V, 1.0 V, respectively), and the electrolysis time of each group was 600 min. The XRD (X-ray diffraction spectrum) patterns of the electrolysis products are depicted in Figure 1a. At a voltage of 0.5 V for the constant cell voltage electrolysis, the primary products of the electrolysis were CaFe_5_O_7_, accompanied by minor quantities of Ca_2_Fe_2_O_5_ and FeO. As the voltage for the constant cell voltage electrolysis experiment was increased, iron oxides were entirely reduced to metallic iron. The heightened driving force led to an accelerated deoxidation rate, with CO_2_ generated by anodic discharge dissolving in the molten salt to form CaCO_3_. With the application of a voltage of 1.0 V for the constant cell voltage electrolysis, Ca_2_Fe_2_O_5_ was completely electrolyzed, and the increased driving force allowed ample time for CO_3_^2−^ ions to diffuse toward the anode, resulting in metallic iron as the ultimate product. According to the experimental results in reference [21], Fe_2_O_3_ is electrolyzed to metal Fe after 600 min at an electrolytic voltage greater than 1.2 V. In comparison, the presence of CaO reduces the electrolytic driving force and speeds up the electrolytic rate. Figure 1b,c shows the SEM and EDS images of the products obtained after 600 min of electrolysis at 1.0 V, which reveal well-defined particle clusters as the predominant components, primarily consisting of metallic iron.

**Figure 2:**
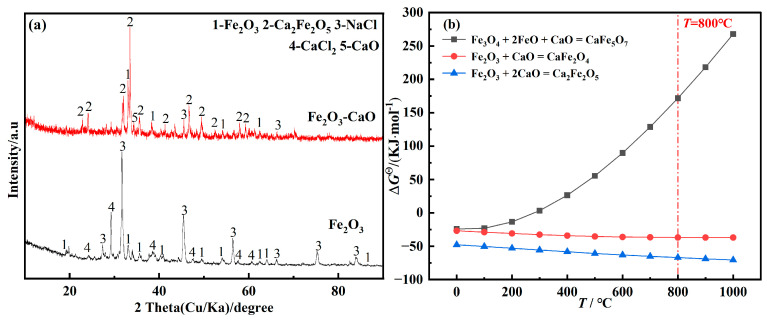
(**a**) XRD patterns of products of Fe_2_O_3_/Fe_2_O_3_-CaO immersed in NaCl-CaCl_2_ molten salt for 10 min; (**b**) temperature dependence of standard Gibbs free energy for the reaction of Fe_2_O_3_ with CaO.

To clarify the phases of Ca_2_Fe_2_O_5_ and CaFe_5_O_7_ generated during constant-voltage electrolysis of Fe_2_O_3_-CaO at 0.5 V, Fe_2_O_3_ and prepared Fe_2_O_3_-CaO cathodes were immersed in NaCl-CaCl_2_ molten salt and maintained for 10 min. The XRD results are shown in Figure 2a. The results indicate that Fe_2_O_3_ does not react with the molten salt without adding CaO. However, when adding CaO, Fe_2_O_3_ primarily forms the new phase Ca_2_Fe_2_O_5_. During the initial stages of constant-voltage piezoelectric electrolysis of Fe_2_O_3_, Ca_2_Fe_2_O_5_ is formed by the chemical reaction between CaO and Fe_2_O_3_ [53]. According to the thermodynamic calculations shown in Figure 2b, it can be inferred that at 800 °C, CaO undergoes a chemical reaction with Fe_2_O_3_ to produce Ca_2_Fe_2_O_5_, while Fe_3_O_4_ does not react with FeO or CaO to form CaFe_5_O_7_. The presence of CaFe_5_O_7_ observed in the XRD results is likely due to the reaction of reduced Fe_3_O_4_ and FeO with CaO at room temperature.

### 2.2. Cyclic Voltammetry Measurements

**Figure 3:**
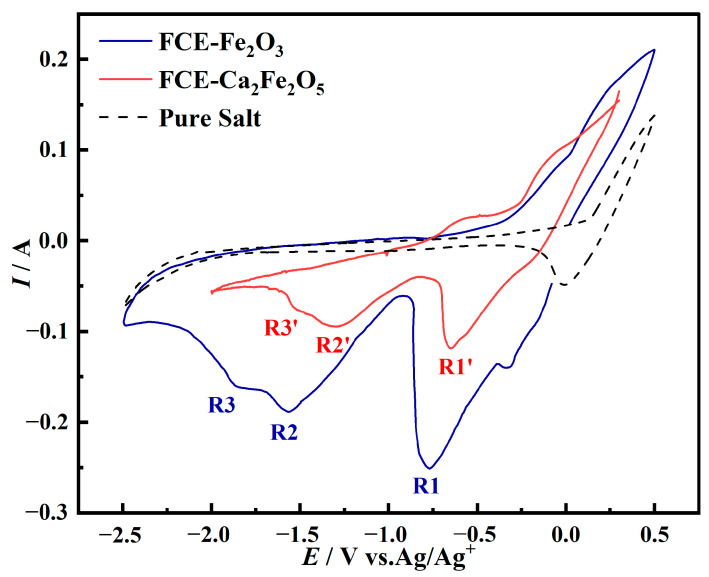
Cyclic voltammetry curves of blank electrodes, FCE-Fe_2_O_3_ and FCE-Ca_2_Fe_2_O_5_ in NaCl-CaCl_2_ molten salt (scan rate: 0.1 V/s, temperature: 800 °C).

Due to the formation of Ca_2_Fe_2_O_5_ from Fe_2_O_3_-CaO at 800 °C, to investigate the electrochemical reduction mechanism of Fe_2_O_3_-CaO, Fe electrodes coated with Fe_2_O_3_ and Ca_2_Fe_2_O_5_ were subjected to cyclic voltammetry tests in a NaCl-CaCl_2_ molten salt at a scan rate of 0.1 V/s, as shown in Figure 3. The dashed line in the figure represents the cyclic voltammetry curve for the blank salt, with a scanning range from −2.5 V to 0.5 V. The solid blue line represents the cyclic voltammetry curve for the FCE-Fe_2_O_3_, and three reduction peaks, denoted as R1, R2, and R3, corresponding to the three-step reduction of Fe, were observed [21]. The solid red line in the figure represents the cyclic voltammetry curve for the FCE-Ca_2_Fe_2_O_5_ with a scanning range from −2.0 V to −0.3 V. The reduction peaks R1’, R2’, and R3’ exhibit an overall positive shift, which is attributed to the faster reaction rate resulting from the addition of CaO. In comparison to FCE-Fe_2_O_3_, the reduction peaks in FCE-Ca_2_Fe_2_O_5_ appear weaker during the scan. This is because of the fact that although the electrode areas are the same, there are fewer active particles participating in the reduction reaction in FCE-Ca_2_Fe_2_O_5_, leading to smaller peak intensities. Additionally, narrowing the scan potential range results in a decrease in peak intensities. Combining XRD phase analysis of the products of Fe_2_O_3_-CaO at different voltages, it can be concluded that the reduction steps of Ca_2_Fe_2_O_5_ are as follows: Ca_2_Fe_2_O_5_ ⟶ Fe_3_O_4_ ⟶ FeO ⟶ Fe.

### 2.3. Constant Voltage Electrolysis and Phase Characterization

**Figure 4:**
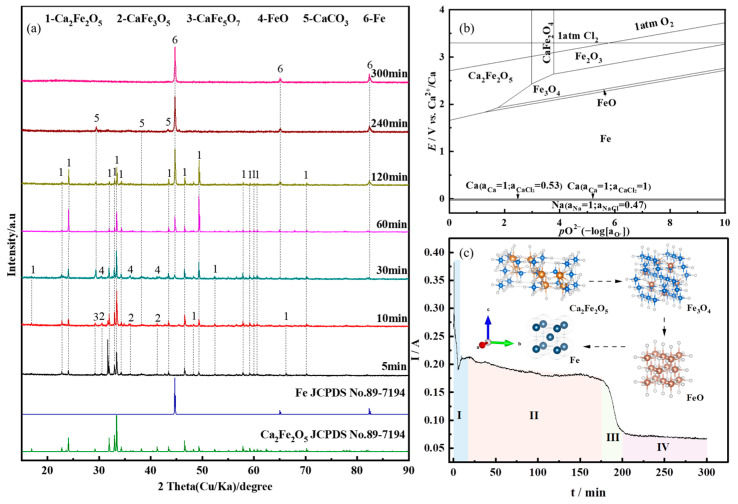
(**a**) XRD patterns of the products of Fe_2_O_3_-CaO in NaCl-CaCl_2_ molten salt under constant voltage electrolysis at 1.0 V for different durations; (**b**) *E*-*p*O_2_ diagram of the Fe-O-Ca system at 800 °C; (**c**) *I*-*t* curve.

To further investigate the phase evolution process of CaO’s reduction of Fe_2_O_3_ during the electrolysis process, and to analyze the deoxidation kinetics, the experiment was conducted at 1.0 V for the constant cell voltage electrolysis for different durations (5~300 min). Figure 4a presents the XRD patterns of the electrolysis products. When the constant cell voltage electrolysis time ranged from 5 min to 10 min, the primary phases detected were Ca_2_Fe_2_O_5_, along with small amounts of CaFe_3_O_5_ and CaFe_5_O_7_. This indicates that under the applied voltage, Fe_2_O_3_ rapidly formed Ca_2_Fe_2_O_5_. At 30 min, FeO and Fe started to appear, while CaFe_3_O_5_ and CaFe_5_O_7_ disappeared. With the extension of the electrolysis time, the diffraction peaks of Ca_2_Fe_2_O_5_ gradually weakened and disappeared at 240 min. Simultaneously, the diffraction peaks of Fe intensified, and at 300 min, Fe was completely reduced to metallic Fe.

**Figure 5:**
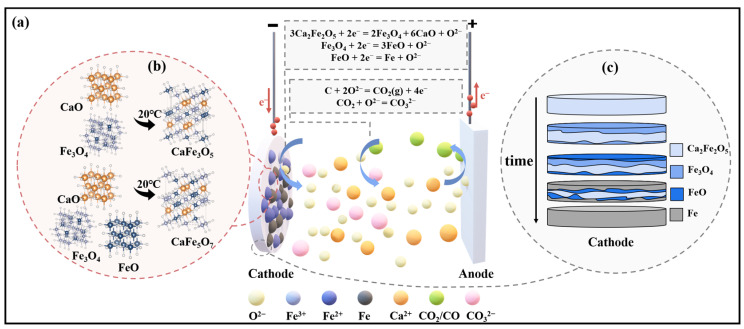
(**a**) Schematic of ion migration during the electrolysis process; (**b**) schematic illustration of intermediate product formation at room temperature; (**c**) diagram depicting the diffusion mechanism at the cathode plate three-phase interface.

During the electrolytic reduction process of Fe_2_O_3_-CaO for iron production, Ca_2_Fe_2_O_5_ is initially formed. The phase evolution process leading to the reduction of metallic iron from Ca_2_Fe_2_O_5_ involves the intermediate phases CaFe_3_O_5_, CaFe_5_O_7_, FeO, and ultimately Fe. Based on the *E*-*p*O_2_ diagram of the Fe-O-Ca system shown in Figure 4b, it can be observed that at certain oxygen partial pressures, the reduction sequence of Ca_2_Fe_2_O_5_ in molten salt is Ca_2_Fe_2_O_5_ ⟶ Fe_3_O_4_ ⟶ FeO ⟶ Fe. When the oxygen partial pressure is low, Fe_3_O_4_ and FeO phases are not detected during the reduction process of Ca_2_Fe_2_O_5_. In Figure 4a, the absence of Fe_3_O_4_ and FeO at 5 min to 10 min of constant cell voltage electrolysis is attributed to the fact that in the early stages of electrolysis, the electrochemical reaction rate is higher than the diffusion rate of O^2−^ to the anode. Consequently, O^2−^ generated during electrolysis accumulates near the cathode. When the sample is removed, O^2−^ forms CaO with Ca^2+^ in the molten salt. At room temperature, CaO reacts with Fe_3_O_4_ to produce CaFe_3_O_5_, and Fe_3_O_4_, FeO and CaO react to produce CaFe_5_O_7_, as shown in Figure 4b. The theoretical decomposition voltage for the reduction of Fe_3_O_4_ (−0.96 V) is lower than that for the reduction of FeO (−1.01 V). As the electrolysis time increases, Fe_3_O_4_ is reduced to FeO, and the phases CaFe_3_O_5_ and CaFe_5_O_7_ disappear. The thermodynamic calculation results of Equations (1), (3) and (5) show that the theoretical decomposition potential of Ca_2_Fe_2_O_5_ is higher than the theoretical decomposition potential of Fe_3_O_4_ and FeO, and the reduction rate of Ca_2_Fe_2_O_5_ is lower than that of Fe_3_O_4_ and FeO. Therefore, after 60~120 min of constant cell voltage electrolysis, the products are mainly Ca_2_Fe_2_O_5_ and Fe. The ion migration during electrolysis is shown in Figure 5a. The reaction process is shown in Equations (1)–(5).

6Ca_2_Fe_2_O_5_ = 4Fe_3_O_4_ + 12CaO + O_2_(g) *E*^⊖^ = −1.49 V, *T* = 800 °C (1)

Fe_3_O_4_ + CaO = CaFe_3_O_5_ Δ*G*^Θ^ = −35.08 kJ·mol^−1^, *T* = 20 °C (2)

2Fe_3_O_4_ = 6FeO + O_2_(g) *E*^⊖^ = −0.96 V, *T* = 800 °C (3)

Fe_3_O_4_ + 2FeO + CaO = CaFe_5_O_7_ Δ*G*^Θ^ = −24.63 kJ·mol^−1^, *T* = 20 °C (4)

2FeO = 2Fe + O_2_(g) *E*^⊖^ = −1.01 V, *T* = 800 °C (5)

When combining the *I*-*t* curve during the electrolysis process, it can be observed that when a voltage is applied to the cathode of Fe_2_O_3_-CaO, there is an initial high current due to the double-layer charging process [36]. In the stage I, the current rapidly decreases, reflecting the fast deoxidation of Ca_2_Fe_2_O_5_ on the cathode surface. As the three-phase reaction interface extends toward the cathode’s interior, the diffusion of O^2−^ to the cathode surface, hindered by the blocking effect, causes fluctuations in the current curve. In the stage Ⅱ, Fe^3+^ gradually reduces to Fe, and dissolved CO_2_ in the molten salt near the cathode reacts with O^2−^ to form CO_3_^2−^, further impeding the diffusion of O^2−^. Consequently, the current decreases. During the stage Ⅲ, an accumulation of O^2−^ near the cathode increases, and the concentration gradient between the cathode and anode accelerates the diffusion rate of O^2−^. The electrochemical rate becomes the limiting step in the reaction, leading to an increase in slope. In the stage Ⅳ, most of the Ca_2_Fe_2_O_5_ has been reduced to Fe, and the reduction in active particles leads to a reduction in the removal of O^2−^ during electrolysis, causing the current curve to level off. Figure 5c illustrates the gradual reduction process of the cathode plate along the three-phase interface towards the interior.

**Figure 6:**
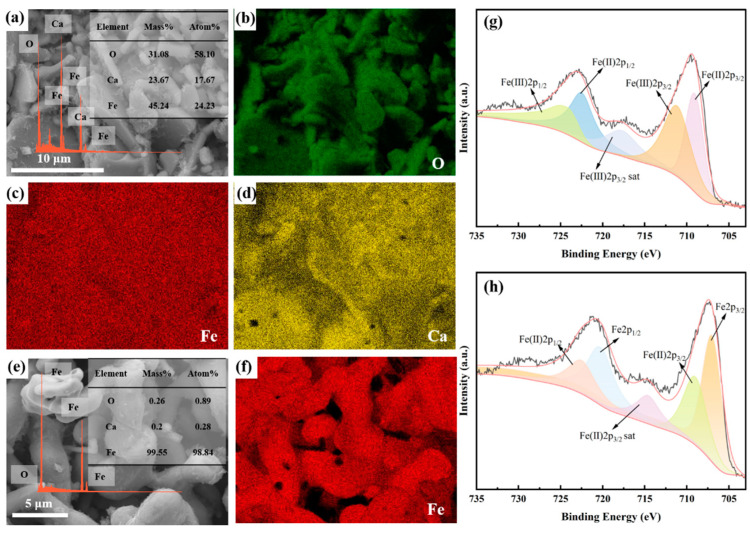
(**a**–**d**) SEM-EDS spectra of the products of Fe_2_O_3_-CaO cathode under 1.0 V electrolysis at 800 °C for 10 min; (**e**,**f**) SEM-EDS spectra of the products of Fe_2_O_3_-CaO cathode under 1.0 V electrolysis at 800 °C for 300 min; (**g**) XPS spectrum of the products obtained after 10 min of electrolysis; (**h**) XPS spectrum of the products obtained after 300 min of electrolysis.

Figure 6 depicts SEM-EDS images and XPS analyses of the products obtained after 10 min and 300 min of constant cell voltage electrolysis. After 10 min of constant cell voltage electrolysis, the products consist of large, flat plate-like particles of Ca_2_Fe_2_O_5_, measuring approximately 10–20 μm in size. Surrounding these, there are smaller particles of CaFe_3_O_5_ and CaFe_5_O_7_, with sizes of around 4–5 μm. Small FeO particles are also observed on the surface of Ca_2_Fe_2_O_5_ particles. The XPS results in Figure 6g indicate that the peaks at binding energies of 709.0 eV and 722.4 eV correspond to Fe(Ⅱ) 2p3/2 and Fe(Ⅱ) 2p1/2, respectively, while the peaks at binding energies of 711.0 eV and 724.4 eV correspond to Fe(Ⅲ) 2p3/2 and Fe(Ⅲ) 2p1/2, respectively. This suggests that some of the Fe(Ⅲ) has been reduced to Fe(Ⅱ) after 10 min of constant cell voltage electrolysis. Upon extending the electrolysis time to 300 min, the products consist of irregular particles with sizes of around 5 μm. Agglomeration between individual grains is observed, as shown in Figure 6e,f. The results in Figure 6h reveal the presence of metallic Fe and Fe(Ⅱ) in the products obtained after 300 min of electrolysis. The presence of metallic Fe aligns with the XRD results, while the appearance of Fe(Ⅱ) can be attributed to the ease of oxidation of metallic iron particles in the presence of air [50].

## 3. Experimental Section and Methods

### 3.1. Materials and Precursor Preparation

**Figure 7:**
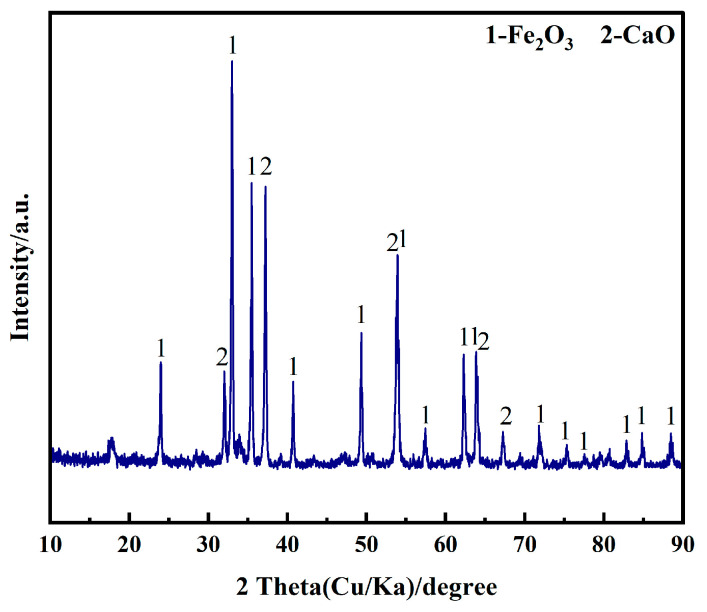
XRD pattern of Fe_2_O_3_-CaO mixed oxides ball-milled for 300 min.

The experiments were carried out by using analytically pure Fe_2_O_3_, CaO, NaCl and CaCl_2_ (Sinopharm Chemical Regent Co., Ltd., Shanghai, China, ≥99.95%). The high-purity graphite sheet (≥99.99%, 15 mm × 100 mm × 3 mm) was polished sequentially on 1000#, 1500#, and 2000# sandpaper to achieve a smooth surface, and its surface was repeatedly washed with deionized water and absolute ethanol. This process aims to minimize the detachment of carbon particles from the surface and prevent short-circuiting during the electrolysis process. A nickel wire (*Φ*1.5 mm) was used to connect the graphite sheet to a stainless steel conductor rod, serving as the anode. Fe_2_O_3_ and CaO (molar ratio 1:2) were mixed with 30 g of anhydrous ethanol in a ball mill. The mixture was blended at 160 r·min^−1^ for 300 min to achieve uniform mixing. The uniformly mixed samples were examined by XRD, and the results are shown in Figure 7. The mixture was then dried at 160 °C for 480 min to completely evaporate the anhydrous ethanol. A total of 1 g of the mixed oxide was taken and compressed into a cylindrical cathode (*Φ*15 mm, 1.9~2.0 mm in thickness) under a pressure of 10 MPa. The cathode was wrapped with a stainless steel mesh (1000#), and a stainless steel wire (*Φ*0.5 mm) was used to connect it to the stainless steel conductor rod, serving as the cathode.

### 3.2. Constant Voltage Electrolysis and Cyclic Voltammetry Testing

**Figure 8:**
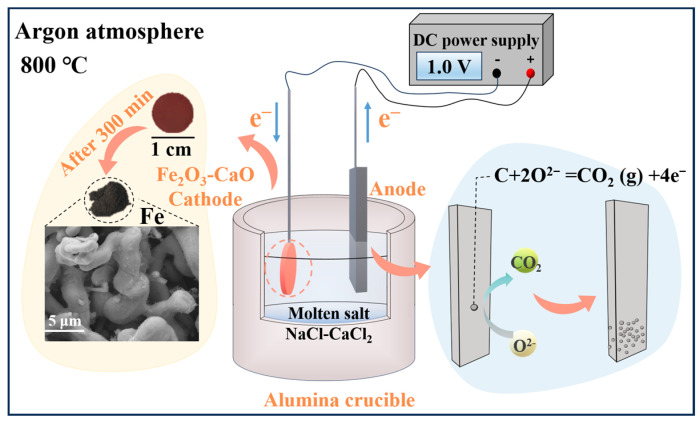
Schematic diagram of electrolysis device.

A molten salt was formulated with a molar ratio of 48:52 (NaCl-CaCl_2_), which had a eutectic point of 504 °C. An electrolysis temperature of 800 °C was chosen to ensure the full melting of NaCl-CaCl_2_ and the low viscosity and fast reaction rate of the molten salt. The mixed molten salt (180 g) was dried at 250 °C for 600 min. The molten salt was heated at a rate of 4 °C/min to the experimental temperature and held for 60 min. Subsequently, a cathode nickel foil (40 mm × 40 mm × 1 mm) and an anode high-purity graphite sheet (120 mm × 40 mm × 5 mm) were used for pre-electrolysis at a voltage of 2.8 V. The reason for pre-electrolysis is to remove the residue moisture and metallic impurities from the molten salt and to decompose oxides and other compounds such as CaOHCl [54]. The experimental process was conducted under argon atmosphere, and the schematic diagram of the electrolysis setup is shown in Figure 8. The constant voltage electrolysis experiment was carried out at 800 °C. After the electrolysis, the obtained cathodic product was subjected to ultrasonic cleaning with deionized water and vacuum drying. Finally, the product was characterized.

Cyclic voltammetry testing was performed using NaCl-CaCl_2_ as the electrolyte at 800 °C. A high-purity graphite sheet served as the auxiliary electrode. A Fe wire (*Φ*1 mm) coated electrode Fe_2_O_3_-CaO and Fe_2_O_3_ (FCE-Ca_2_Fe_2_O_5_, FCE-Fe_2_O_3_) was used as the working electrode. An Ag/Ag^+^ reference electrode was prepared by filling a mullite tube (*Φ*5 mm) with a molar ratio of NaCl:CaCl_2_:AgCl = 49:49:2 and silver wire (*Φ*0.5 mm, 99.99%). The experiments were conducted under argon atmosphere.

### 3.3. Characterization

The X-ray diffraction spectrum (XRD, X-ray 6000 with Cu Kα1 radiation λ = 1.5405 Å, scanning speed 10 °/min, Rigaku Corporation, Tokyo, Japan) was used for phase detection of the cathodic product. The microscopic morphology (SEM, JEM-2900F, Japan Electronics Co., Ltd., Tokyo, Japan) and elemental composition of the product were analyzed using a scanning electron microscope and an energy dispersive spectroscopy (EDS). X-ray photoelectron spectroscopy (XPS, Ulvac-Phi, Chigasaki, Japan) was employed to obtain information about the types of elements, the binding states of material atoms, and the distribution of charges. The equipment used for electrochemical testing was an electrochemical workstation model CHI660e (Shanghai Chenhua Co., Ltd., Shanghai, China).

## 4. Conclusions

By adding CaO to Fe_2_O_3_ for the preparation of a metal oxide cathode, two-electrode electrolysis experiments were conducted in NaCl-CaCl_2_ molten salt at 800 °C. The influence of different voltages and durations on the phase composition, morphology, and valence state of the electrolysis products was studied. Combined with three-electrode cyclic voltammetry tests, the electrochemical deoxidation and reduction mechanisms of Fe_2_O_3_ in the presence of CaO were analyzed, leading to the following conclusions:

(1) In NaCl-CaCl_2_ molten salt, Fe_2_O_3_ and CaO can spontaneously undergo a chemical reaction to generate Ca_2_Fe_2_O_5_ at 800 °C without applying electrolytic voltage. The reduction steps of Ca_2_Fe_2_O_5_ are Ca_2_Fe_2_O_5_ ⟶ Fe_3_O_4_ ⟶ FeO ⟶ Fe. Compared to the reduction of Fe_2_O_3_ alone, the addition of CaO reduces the electrolysis voltage required for the reduction to metallic Fe, facilitating the progress of the electrolysis process. Metallic Fe and CaCO_3_ can be generated after electrolysis at 0.6 V for 600 min.
(2) Under the condition of 1 V electrolysis, the cathodic deoxidation process of Fe_2_O_3_-CaO for the production of metallic iron results in the formation of CaFe_3_O_5_, CaFe_5_O_7_, FeO, and Fe phases over time. The appearance of CaFe_3_O_5_ and CaFe_5_O_7_ phases is attributed to the interaction of Fe_3_O_4_, Ca^2+^, and O^2−^ generated during the electrolysis process, as well as the subsequent reaction of Fe_3_O_4_, FeO, Ca^2+^, and O^2−^ at room temperature. Under the high driving force of 1 V, the reaction time for the electrolysis of Fe_2_O_3_-CaO to Fe is reduced, and after 300 min of electrolysis, it is entirely converted to metallic Fe.

## Data Availability

Data is contained within the article.

## References

[1] Yu X., Tan C. (2022). China’s pathway to carbon neutrality for the iron and steel industry. Glob. Environ. Chang..

[2] Wang Y.X., Liu J., Tang X.L., Wang Y., An H.W., Yi H.H. (2023). Decarbonization pathways of China’s iron and steel industry toward carbon neutrality. Resour. Conserv. Recycl..

[3] Chang Y.F., Wan F., Yao X.L., Wang J.X., Han Y.F., Li H. (2023). Influence of hydrogen production on the CO_2_ emissions reduction of hydrogen metallurgy transformation in iron and steel industry. Energy Rep..

[4] Liu L.T., Zhai R.R., Hu Y.D. (2023). Performance evaluation of wind-solar-hydrogen system for renewable energy generation and green hydrogen generation and storage: Energy, exergy, economic, and enviroeconomic. Energy.

[5] Al-Ghussain L., Ahmad A.D., Abubaker A.M., Hassan M.A. (2022). Exploring the feasibility of green hydrogen production using excess energy from a country-scale 100% solar-wind renewable energy system. Int. J. Hydrogen Energy.

[6] Najjar Y.S. (2013). Hydrogen safety: The road toward green technology. Int. J. Hydrogen Energy.

[7] Acar C., Dincer I. (2019). Review and evaluation of hydrogen production options for better environment. J. Clean. Prod..

[8] Wang Z., Zhang X., Rezazadeh A. (2021). Hydrogen fuel and electricity generation from a new hybrid energy system based on wind and solar energies and alkaline fuel cell. Energy Rep..

[9] Tang J., Chu M.S., Li F., Feng C., Liu Z.G., Zhou Y.S. (2020). Development and progress on hydrogen metallurgy. Int. J. Miner. Metall. Mater..

[10] Sun L., Yin J., Bilal A.R. (2023). Green financing and wind power energy generation: Empirical insights from China. Rebew. Energy.

[11] Nian V., Chou S.K. (2014). The state of nuclear power two years after Fukushima–The ASEAN perspective. Appl. Energy.

[12] Nian V., Mignacca B., Locatelli G. (2014). Policies toward net-zero: Benchmarking the economic competitiveness of nuclear against wind and solar energy. Appl. Energy.

[13] Allanore A. (2014). Features and challenges of molten oxide electrolytes for metal extraction. J. Electrochem. Soc..

[14] Allanore A., Lavelaine H., Valentin G., Birat J.P., Lapicque F. (2008). Iron Metal Production by Bulk Electrolysis of Iron Ore Particles in Aqueous Media. J. Electrochem. Soc..

[15] Tang D., Yin H., Xiao W., Zhu H., Mao X., Wang D. (2013). Reduction mechanism and carbon content investigation for electrolytic production of iron from solid Fe_2_O_3_ in molten K_2_CO_3_–Na_2_CO_3_ using an inert anode. J. Electroanal. Chem..

[16] Sadoway D.R. (1995). New opportunities for metals extraction and waste treatment by electrochemical processing in molten salts. J. Mater. Res..

[17] Zhang K., Jiao H., Zhou Z., Jiao S., Zhu H. (2016). Electrochemical behavior of Fe (III) ion in CaO-MgO-SiO_2_-Al_2_O_3_-NaF-Fe_2_O_3_ melts at 1673 K. J. Electrochem. Soc..

[18] Chen G.Z., Fray D.J., Farthing T.W. (2000). Direct electrochemical reduction of titanium dioxide to titanium in molten calcium chloride. Nature.

[19] Xi X.L., Feng M., Zhang L.W., Nie Z.R. (2020). Applications of molten salt and progress of molten salt electrolysis in secondary metal resource recovery. Int. J. Miner. Metall. Mater..

[20] Li M., Liu C.Y., Ding A.T., Xiao C.L. (2023). A review on the extraction and recovery of critical metals using molten salt electrolysis. J. Environ. Chem. Eng..

[21] Li H., Jia L., Liang J.L., Yan H.Y., Cai Z.Y., Reddy R.G. (2019). Study on the Direct Electrochemical Reduction of Fe_2_O_3_ in NaCl-CaCl_2_ Melt. Int. J. Electrochem. Sci..

[22] Li H., Jia L., Cao W.G., Liang J.L., Wang L., Yan H.Y. (2021). The electrochemical reduction mechanism of Fe_3_O_4_ in NaCl-CaCl_2_ melts. Int. J. Chem. React. Eng..

[23] Fray D., Schwandt C. (2017). Aspects of the application of electrochemistry to the extraction of titanium and its applications. Mater. Trans..

[24] Chen G.Z., Gordo E., Fray D.J. (2004). Direct electrolytic preparation of chromium powder. Metall. Mater. Trans. B.

[25] Liu Z.W., Zhang H.L., Pei L.L., Shi Y.L., Cai Z.H., Xu H.B., Zhang Y. (2018). Direct electrolytic preparation of chromium metal in CaCl_2_–NaCl eutectic salt. Trans. Nonferrous Met. Soc. China.

[26] Huan S.X., Wang Y.W., Peng J.P., Di Y.Z., Li B., Zhang L.D. (2020). Recovery of aluminum from waste aluminum alloy by low-temperature molten salt electrolysis. Miner. Eng..

[27] Chen Y.F., Wang M.Y., Lv A.J., Zhao Z.H., An J.L., Zhang J.T., Tu J.G., Jiao S.Q. (2021). Green preparation of vanadium carbide through one-step molten salt electrolysis. Ceram Int..

[28] An J.L., Wang M.Y., Jia Y.Z., Chen Y.F., Jiao S.Q. (2022). Facile preparation of metallic vanadium from consumable V_2_CO solid solution by molten salt electrolysis. Sep. Purif. Technol..

[29] Chang C., Tu J.G., Chen Y.F., Wang M.Y., Jiao S.J. (2022). Electro-deoxidation behavior of solid SeO_2_ in a low-temperature molten salt. Chem. Commun..

[30] Zhu X., Li L., Song W.C., Zhang D.F., Ma S.R., Qiu K.H. (2021). Electrochemical synthesis of Ti–Al–V alloy by chlorination of Ti_2_O_3_ and V_2_O_3_ in AlCl_3_-containing molten chloride salt. J. Mater. Res. Technol..

[31] Cao X.Z., Li Q.Y., Shi Y.Y., Wu D., Xue X.X. (2022). Preparation of V-4Cr-4Ti Alloys from Mixed Oxides via Electro-Deoxidation Process in Molten Salt. Metals.

[32] Li S.S., Zou X.L., Zheng K., Lu X.G., Chen C.Y., Li X., Xu Q., Zhou Z.F. (2018). Electrosynthesis of Ti_5_Si_3_, Ti_5_Si_3_/TiC and Ti_5_Si_3_/Ti_3_SiC_2_ from Ti-bearing blast furnace slag in molten CaCl_2_. Metall. Mater. Trans. B.

[33] Sure J., Vishnu D.S.M., Kumar R.V., Schwandt C. (2019). Molten salt electrochemical synthesis, heat treatment and microhardness of Ti-5Ta-2Nb alloy. Mater. Trans..

[34] Hu D., Xiao W., Chen G.Z. (2013). Near-net-shape production of hollow titanium alloy components via electrochemical reduction of metal oxide precursors in molten salts. Metall. Mater. Trans. B.

[35] Yang X., Jiao H., Wang M.Y., Jiao S.Q. (2019). Direct preparation of V–Al alloy by molten salt electrolysis of soluble NaVO_3_ on a liquid Al cathode. J. Alloys Compd..

[36] Zhua F.X., Li K.H., Song W.C., Li L., Zhang D.F., Qiu K.H. (2021). Composition and structure of Ti–Al alloy powders formed by electrochemical co-deposition in KCl–LiCl–MgCl_2_–TiCl_3_–AlCl_3_ molten salt. Intermetallics.

[37] Sure J., Vishnu D.S.M., Schwandt C. (2017). Direct electrochemical synthesis of high-entropy alloys from metal oxides. Appl. Mater. Today.

[38] Schwandt C., Doughty G.R., Fray D.J. (2010). The FFC-Cambridge Process for Titanium Metal Winning.

[39] Jiang K., Hu X., Ma M., Wang D., Qiu G., Jin X., Chen G.Z. (2006). “Perovskitization”-Assisted electrochemical reduction of solid TiO_2_ in Molten CaCl_2_. Angew. Chemie..

[40] Descallar-Arriesgado R.F., Kobayashi N., Kikuchi T., Suzuki R.O. (2011). Calciothermic reduction of NiO by molten salt electrolysis of CaO in CaCl_2_ melt. Electrochim. Acta.

[41] Schwandt C. (2018). On the nature of the current and the absence of an IR-drop in an FFC-Cambridge-type electro-deoxidation cell. Electrochim. Acta.

[42] Pang Z.Y., Li X., Zhang X.Q., Li J.J., Wang S.J., Xiong X.L., Li G.S., Qian. X., Zhou Z.F., Zou X.L. (2022). Molten salt electrosynthesis of silicon carbide nanoparticles and their photoluminescence property. Trans. Nonferrous Met. Soc. China.

[43] Schwandt C., Fray D.J. (2005). Determination of the kinetic pathway in the electrochemical reduction of titanium dioxide in molten calcium chloride. Electrochim. Acta.

[44] Liang J.L., Wang D.B., Wang L., Li H., Cao W.G., Yan H.Y. (2022). Electrochemical process for recovery of metallic Mn from waste LiMn_2_O_4_-based Li-ion batteries in NaCl−CaCl_2_ melts. Int. J. Min. Met. Mater..

[45] Li H., Xue C.L., Yang Y., Liang J.L. (2023). Preparation of Fe_3_Si and FeSi intermetallic compounds from copper slag by electrochemical method. J. Iron Steel Res. Int..

[46] Zhou Z.G., Hua Y.X., Xu C.Y., Li J., Li Y., Zhang Q.B., Zhang Y.D., Kuang W.H. (2016). Synthesis of micro-FeTi powders by direct electrochemical reduction of ilmenite in CaCl_2_-NaCl molten salt. Ionics.

[47] Wu T., Ma X., Jin X. (2016). Preparation of vanadium powder and vanadium-titanium alloys by the electroreduction of V_2_O_3_ and TiO_2_ powders. Mater. Res..

[48] Abdelkader A.M., Fray D.L. (2012). Electro-deoxidization of hafnium dioxide and niobia-doped hafnium dioxide in molten calcium chloride. Electrochim. Acta.

[49] Lee M.J., Noh J.S., Kim K.Y., Lee J.H. (2014). Electrochemical deoxidization of ZrSiO_4_ in molten calcium chloride. Phys. Met. Metallogr..

[50] Bagus P.S., Nelin C.J., Brundle C.R., Vincent Crist B., Lahiri N., Rosso K.M. (2021). Combined multiplet theory and experiment for the Fe 2p and 3p XPS of FeO and Fe_2_O_3_. Chem. Phys..

[51] Qu Z.F., Hu M.L., Gao L.Z., Lai P.S., Bai C.G. (2018). Preparation of Titanium Foams Through Direct Electrolysis of the Sintered CaO-TiO_2_ in Molten Salt CaCl_2_. 9th International Symposium on High-Temperature Metallurgical Processing.

[52] Xu Y., Zhao G., Cai Y. (2021). Preparation of Titanium by Electro-deoxidation of CaTiO_3_ in a Molten CaCl_2_-NaCl Salt. Int. J. Electrochem. Sci..

[53] Chen G.Z., Fray D.J. (2004). Understanding the electro-reduction of metal oxides in molten salts. Light Metals-Warrendale-Proceedings.

[54] Yan X.Y., Fray D.J. (2002). Production of niobium powder by direct electrochemical reduction of solid Nb_2_O_5_ in a eutectic CaCl_2_-NaCl melt. Metall. Mater. Trans. B.

